# Late Effects of ^1^H + ^16^O on Short-Term and Object Memory, Hippocampal Dendritic Morphology and Mutagenesis

**DOI:** 10.3389/fnbeh.2020.00096

**Published:** 2020-06-26

**Authors:** Frederico Kiffer, Tyler Alexander, Julie Anderson, Thomas Groves, Taylor McElroy, Jing Wang, Vijayalakshmi Sridharan, Michael Bauer, Marjan Boerma, Antiño Allen

**Affiliations:** ^1^Division of Radiation Health, University of Arkansas for Medical Sciences, Little Rock, AR, United States; ^2^Department of Pharmaceutical Sciences, University of Arkansas for Medical Sciences, Little Rock, AR, United States; ^3^Neurobiology & Developmental Sciences, University of Arkansas for Medical Sciences, Little Rock, AR, United States; ^4^Department of Biomedical Informatics, University of Arkansas for Medical Sciences, Little Rock, AR, United States

**Keywords:** Mars, brain, neuron, hippocampus, morphology, behavior, space, radiation

## Abstract

The space extending beyond Earth’s magnetosphere is subject to a complex field of high-energy charged nuclei, which are capable of traversing spacecraft shielding and human tissues, inducing dense ionization events. The central nervous system is a major area of concern for astronauts who will be exposed to the deep-space radiation environment on a mission to Mars, as charged-particle radiation has been shown to elicit changes to the dendritic arbor within the hippocampus of rodents, and related cognitive-behavioral deficits. We exposed 6-month-old male mice to whole-body ^1^H (0.5 Gy; 150 MeV/n; 18–19 cGy/minute) and an hour later to ^16^O (0.1Gy; 600 MeV/n; 18–33 Gy/min) at NASA’s Space Radiation Laboratory as a galactic cosmic ray-relevant model. Animals were housed with bedding which provides cognitive enrichment. Mice were tested for cognitive behavior 9 months after exposure to elucidate late radiation effects. Radiation induced significant deficits in novel object recognition and short-term spatial memory (Y-maze). Additionally, we observed opposing morphological differences between the mature granular and pyramidal neurons throughout the hippocampus, with increased dendritic length in the dorsal dentate gyrus and reduced length and complexity in the CA1 subregion of the hippocampus. Dendritic spine analyses revealed a severe reduction in mushroom spine density throughout the hippocampus of irradiated animals. Finally, we detected no general effect of radiation on single-nucleotide polymorphisms in immediate early genes, and genes involved in inflammation but found a higher variant allele frequency in the antioxidants thioredoxin reductase 2 and 3 loci.

## Introduction

NASA’s efforts to extend manned spaceflight beyond low-earth orbit in the near future for the first time since the Apollo era dawns a new age in space exploration. Current plans involve lunar sortie missions followed by the deep-space gateway, where astronaut crews will be exposed to radiation, confinement, and microgravity for increasing durations-all leading to a manned mission to Mars, targeted toward the late 2030s (Drake et al., 2010). This new age in space exploration brings new challenges for long-term life support as humans have never been exposed to the complex radiation environment of deep space for more than 13 days.

The interplanetary space within the solar system is host to high-energy charged particles that consist of galactic cosmic rays (GCR), and solar particle events (SPE), such as coronal mass ejections. Galactic cosmic rays originate from supernovas within our galaxy, and provide constant fluence of approximately 87% ^1^H, 12% ^4^He, and 1–2% high-mass (Z > 2) high-energy (HZE) particles at median energies of 1,000 MeV/n (Nelson, 2016). Of all HZE, ^16^O is the most abundant particle, and is encountered at median energies of approximately 600 MeV/n (George et al., 2009). Similarly, solar particle events deliver almost exclusively ^1^H and ^4^He, but unlike the constant GCR fluence, SPE exposures are dependent upon the 11-year solar cycle, and greatly vary in energies (NCRP, 2006). In addition, the increased heliospheric magnetic fields observed during solar maximum act to “shield” incoming GCR-effectively reducing their fluence, by approximately one order of magnitude (Nelson, 2016). Because of these phenomena, predicting the exact dosage encountered on a mission to mars is not tangible. The dose estimates defined by the current Mars design reference architecture are between 0.25 and 0.5 Gy for GCR, with an additional 0.15–0.5 Gy for shielded exposures to SPE (Drake et al., 2010). Protons deliver approximately 50–60%, neutrons 10–20%, and HZE particles 10–20% of the relative total organ dose (Gy) from GCR (Nelson et al., 2016).

There is currently no feasible shielding capable of mitigating all high-energy charged particles. In addition, traditional aluminum shields may increase total body dosage due to particle fragmentation and back-scatter within the spacecraft (Cucinotta et al., 2012). The stochastic nature of particle interactions also adds uncertainty to dosage predictions. The central nervous system (CNS) may be particularly vulnerable to charged-particle radiation due to the discriminate regions in which neurogenesis occurs and the permanence of the G_0_ phase in mature neurons. Recent *in silico* studies suggest that the dendrites of hippocampal neurons are particularly likely to undergo primary ionization events in response to charged-particle radiation, and have found that dendrites are 10-times more likely to be penetrated by charged-particles than the soma (Nelson et al., 2016). The added range in which incident δ-rays are capable of reaching-up to 1 cm-further exacerbates the likelihood of ionization events within dendrites (Alp and Cucinotta, 2018). NASA’s concern for radiation-induced CNS insults include possible in-flight alterations to cognitive and motor function, and late neurological pathologies, including Alzheimer’s disease, dementia, and premature aging (Nelson et al., 2016). Additionally, the National Council for Radiation Protection and Measurements recognizes that there may be subclinical CNS symptoms capable of compromising mission success (on Radiation Protection and (NCRP, 2016).

The effects of high-energy ^1^H and HZE particles at doses below 1 Gy on hippocampus-dependent cognition have produced a body of literature describing cognitive-behavioral deficits in mice and rats (Kiffer et al., 2019b). High-energy ^1^H exposures have likewise been overwhelmingly known to induce deficits in hippocampus-dependent rodent behavior at low doses (Kiffer et al., 2019b). Recent particle accelerator advancements at NASA’s Space Radiation Laboratory have allowed for multiple particle exposures, subsequently improving space radiation simulations. At this time few CNS studies have subjected animals to multiple space-relevant particle exposures (Raber et al., 2016, 2019; Kiffer et al., 2018; Krukowski et al., 2018). The study by Krukowski et al. (2018) observed that exposures of 0.1 or 0.5 Gy of ^1^H + ^4^He + ^16^O resulted in dose- and sex-dependent deficits in hippocampus-dependent Novel Object Recognition (NOR), sociability, social memory, and anxiety. However, the recent study by Raber and colleagues reveals 0.5 or 2 Gy of ^1^H + ^16^O + ^28^Si resulted in NOR deficits in male and female B6D2F1 mice (Raber et al., 2019). We have previously shown that a 0.6 Gy exposure to ^1^H + ^16^O within the same day induced short-term spatial memory deficits, as assessed by the Y-maze 3 months after exposure, and altered dendritic morphology in the hippocampus of male C57Bl/6J mice (Kiffer et al., 2018). The present study explores the effects on hippocampus-dependent behaviors 9 months after a 0.6 Gy exposure to ^1^H + ^16^O.

## Materials and Methods

### Animals and Irradiation

Male C57Bl/6J mice were acquired from Jackson Laboratories (Bar Harbor, ME, United States) and housed five per cage. Animals received water and standard low-soy rodent chow (2020X; Harlan^®^ Laboratories Inc; Indianapolis, IN, United States) *ad libitum* and were housed on a 12:12 h light-dark schedule for the duration of the study. Mice were transported to Brookhaven National Laboratory, Upton, NY, by overnight airlift at 6-months of age. After 1-week of acclimation, mice received whole-body irradiation at the NASA Space Radiation Laboratory. During irradiation, mice were placed in individual well-ventilated Lucite holders, five at a time, and holders were mounted perpendicular to the beam direction. Animals first received 0.5 Gy of ^1^H (150 MeV/n; 18–19 cGy/minute) and were placed back in their cages. Approximately 1 h later, mice were placed back in the Lucite holder and onto the beam line and exposed to 0.1 Gy ^16^O (600 MeV/n; 18–33 cGy/minute). Dosimetry and beam properties were controlled by NASA Space Radiation Laboratory physicists. Sham-irradiated animals were placed on the beam line but did not receive charged-particle radiation. After irradiation, animals were transported back to the University of Arkansas for Medical Sciences (UAMS) by overnight airlift and given 2020X chow containing 150 ppm fenbendazole for 8 weeks, as a routine UAMS quarantine procedure. Mice remained housed five per cage with Enrich-o’Cob^®^ (The Andersons, Inc) bedding. All animal procedures were approved by the Institutional Animal Care and Use Committees of UAMS and Brookhaven National Laboratory.

### Y-Maze

Animal behavior was tested 9 months after irradiation (*n* = 10 per treatment). Mice were first tested via the Y-maze, which relies on the animals’ endogenous drive for exploring spatial novelty, not on negative or positive reinforcement (Dellu et al., 1992). The Y-maze is constructed out of opaque acrylic and consists of three similar arms (45 cm × 15 cm × 30 cm): start, familiar, and novel. The familiar and novel arms contain an object of different size and shape mounted at the end of the arm, with no other obvious environmental cues (other than the ceiling mounted camera). Animals are placed in the start arm facing away from the center of the maze, and the familiarization session consists of free exploration of the start and familiar arms. For the testing session, animals were again placed in the maze 3 h after the familiarization session, but this time with access to all arms. Allocation of arms (start, familiar, novel) was counterbalanced between and within each experimental group. Trials lasted for 5 min, and center and nose-points were recorded throughout each session. All experimental arenas were wiped clean with 20% EtOH solution after each trial. Behavioral experiments were recorded on a charge-coupled video camera, located above the maze for automatic behavioral analysis with EthoVision software version 11 (Noldus Information Technology).

### Novel Object Recognition

NOR testing relies on animals’ endogenous propensity for exploring novelty in their environment; unimpaired animals will spend significantly more time exploring the novel object. After the Y-maze testing, animals were tested for NOR, a 4-day procedure in which animals freely explore an arena for 10 min each day. The first 2 days serve as habituation days, in which mice are able to explore an empty arena, effectively serving as an open field test; locomotor activity is measured at this stage. Familiarization occurs on day three, when animals explore an arena containing two identical objects (cell-culture flasks filled with sand). Novel object recognition testing occurs on day four, when one of the now familiar objects is replaced by a novel object (large LEGO^®^ blocks assembled to a similar size as the cell-culture flasks) (Leger et al., 2013). The arena is a 40 cm × 40 cm × 40 cm cube consisting of an aluminum floor, non-transparent acrylic walls, and an open ceiling. Animals were placed in the center of the arena parallel to the objects to ensure no bias. The tracking software was programmed to track animal-center points for the habituation trials and the animal-nose points during familiarization and testing trials.

### Golgi Staining

Shortly after behavioral testing, animals were anesthetized with isofluorane, exsanguinated via the inferior vena cava, and their brains were extracted and dissected along the midsagittal plane. Golgi staining is a widely accepted and reliable method for assessing dendrite and dendritic spine dynamics in response to various treatments due to the low percentage of random neuron impregnation, which allows for low background noise, and resistance to fading or photobleaching over time (Mikolaenko et al., 2005; Giddabasappa et al., 2011). We adapted a staining protocol and used the reagents contained in the superGolgi kit (Bioenno Tech), as previously described (Groves et al., 2017). All morphological and molecular experiments were performed in animals that underwent behavioral testing. A total of five brains from each treatment group, and the right hemispheres were stained.

### Dendritic Morphology Quantification and Spine Analyses

We analyzed dendritic spines of coded, Golgi-impregnated dorsal and ventral hippocampus sections. Molecular layer dendrites from dorsal Dentate granular neurons were traced. We also examined dendrites in the apical (*stratum radiatum*) and basal (*stratum oriens*) divisions of pyramidal neurons of the dorsal CA1, and ventral CA3 subregions. Neurons meeting the following criteria were suitable for analyses: (1) presence of well-defined, non-truncated dendrites respective to the coronal plane, (2) consistently dark Golgi staining along the entire extent of the dendrite, and (3) relative isolation from neighboring neurons to avoid interference with analysis. Five dendritic segments (each at least 20 μm long) per granular, or pyramidal neuron were analyzed, and six to seven neurons were analyzed per brain (Titus et al., 2007; Magariños et al., 2011). Neurons that met staining criteria were traced using a 60 X objective, (100 X oil objective for spine analyses), a computerized oscillating stage for 3-dimensional imaging, and Neurolucida software (Ver. 11, Microbrightfield, Inc., Williston, VT, United States).

We quantified the morphology of the granular and pyramidal neurons contained in the hippocampal formation by Sholl analysis, total dendritic length, number of branch points, and dendritic complexity index (DCI) with the NeuroExplorer component of the Neurolucida program. Sholl analysis is used to assess the amount and distribution of the arbor at increasing radial distances from the cell body (Sholl, 1953). Radii were set to extend in 10 μm intervals from the soma. The length of each dendritic branch, within each progressively larger circle, was counted from the soma, with respect to three dimensions.

Next, we performed branch-point analyses, a method of quantifying the number of bifurcations and the order in which they occur (Morley and Mervis, 2013). Lower branch-point orders represent proximal regions of the arbor, whereas higher branch-point orders characterize distal regions. We used branch-point analysis to determine the complexity of dendritic arborization, because the complexity of the dendritic tree is an important phenotypic component of branching analysis and provides insight into circuit modulation. The DCI was determined by the following equation:

$$\;\mathrm{DCI}=\sum \left(\left(\mathrm{branch}\,\mathrm{tip}\,\mathrm{orders}+\mathrm{branch}\,\mathrm{tips}\right)\times \left(\frac{\mathrm{total}\,\mathrm{dendritic}\,\mathrm{length}}{\mathrm{primary}\,\mathrm{dendrites}}\right)\right)$$

In the *cornu ammonis* (CA) 1 and CA3 subregions of the hippocampus, apical and basal dendrites were analyzed separately. We traced five randomly stained neurons per subregion per animal. Morphological experiments were conducted blinded to treatment groups.

### DNA Extraction and Quality Control

At the time of sacrifice, left brain hemispheres were dissected, and the hippocampi were promptly collected and fresh frozen in liquid nitrogen prior to storage in -80°C. gDNA was extracted with the All-Prep extraction kit by MIDSCI^®^ in accordance to the manufacturer’s protocol. Nucleic acid purity and concentration were determined by Nanodrop 2000^TM^ spectrophotometry and Qubit^TM^ fluorometry (Thermo Fisher Scientific^TM^).

### DNA Sequencing and Single-Nucleotide Polymorphism (SNP) Analyses

gDNA for all samples (*n* = 10) was normalized to 10 ng/uL and prepared for sequencing according to the Truseq custom amplicon low input library preparation protocol (Illumina^®^ document 1000000002191 v04). Library qualities were assessed via Advanced Analytical^TM^ fragment analyses (Agilent^®^). Pooled libraries were prepared for next-generation targeted sequencing in accordance to the manufacturer’s protocol (Illumina^®^ MiniSeq Denature and Dilute Guide, document 1000000002697 v00). PhiX v3 (Illumina^®^) was used as an assay control. Overall run quality and individual reads were evaluated and filtered based on the manufacturer’s criteria, and *post hoc* quality filters: Variant is on target, loci or site genotypes were not in regions with conflicting indel calls, GQX (genotype quality minimum) and GQ (genotype quality) is greater than 30, Locus QD (quality by depth/variant confidence) is generally greater than two (in low QD instances, mapping quality is first checked and allelic depths are individually considered), and MQ (root mean square of the mapping quality of the call) is above 20.

A proportion of gene targets in our panel involved only coding regions (CDS) whereas a proportion involved full target regions, including untranslated regions (Supplementary Table 1). In all cases, known common SNPs in accordance with the NCBI37/mm9 genome assembly, as compiled by MGI dbSNP (build 142), were omitted from analyses. Previously unidentified SNPs, which were identical and present in all or most samples have likewise been omitted from analyses (Supplementary Table 2).

### Statistical Analyses

We expressed data as a mean ± the standard error of the mean (SEM). Analysis of behavioral data occurred throughout the length of each test. Discrimination ratios (DR) were calculated as:

$$\mathrm{DR}=\frac{\left(\mathrm{novel}\,\mathrm{exploration}-\mathrm{familiar}\,\mathrm{exploration}\right)}{\left(\mathrm{novel}\,\mathrm{exploration}+\mathrm{familiar}\,\mathrm{exploration}\right)}$$

Behavioral assays comparing discrimination ratios or object exploration in individual treatment groups were analyzed via unpaired *t*-tests. We used a one-way analysis of variance (ANOVA) followed by *post hoc* multiple comparisons with Bonferroni corrections to evaluate statistical differences within sham and irradiated groups in measures of time spent in Y-maze arms. Sholl analyses were conducted via two-way ANOVA to test for effects of treatment and distance from the soma, with a *post hoc* multiple comparisons corrected by Fisher least-significant-difference (LSD), when appropriate. A Repeating Measures ANOVA was used to assess main effects of radiation on habituation learning, with test day being the repeated measure, and a Tukey correction for *post hoc* multiple comparisons. We employed unpaired *t-*tests with Welch’s corrections to evaluate differences in dendritic complexity index, spine density, and variant allele frequency between sham and irradiated mice. Statistical analyses for behavioral, morphological and sequencing assays were conducted with GraphPad Prism 8.0 software (La Jolla, CA, United States) and *P* < 0.05 was considered significant.

## Results

### Behavior

**FIGURE 1:**
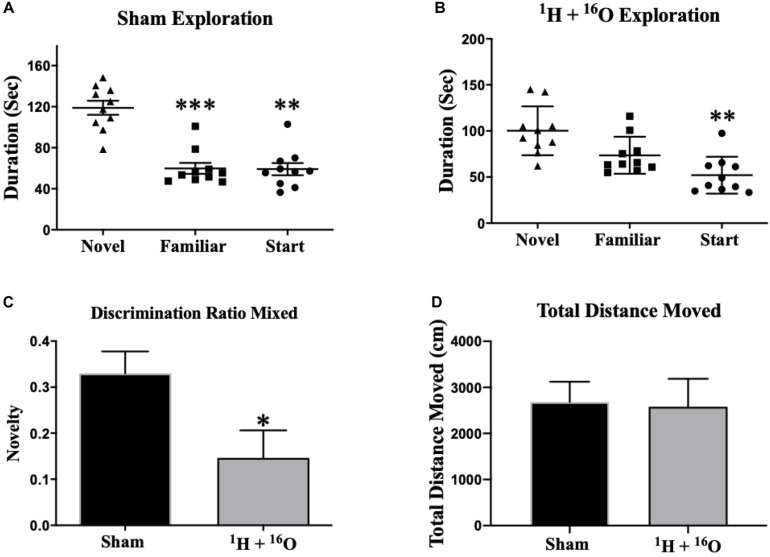
Y-maze. **(A)** Sham-irradiated mice explored the novel arm for a significantly longer duration than the other arms during testing, indicating normal short-term spatial memory. **(B)** Irradiated animals spent more time exploring the novel than the start arm, but they were unsuccessful in exploring the novel arm for a longer duration than the familiar arm, implying spatial memory injury. **(C)** Discrimination ratios for both treatment groups reveal a marked decrease in novel arm discrimination for irradiated animals. **(D)** There was no effect of radiation on gross locomotor activity based on mean total distances moved. Average ± SEM (*n* = 10); *P < 0.05, **P < 0.01, ***P < 0.001.

Y-maze performance is contingent upon animals’ ability to retain spatial memory encoded in the familiarization phase of the trial and recall it during the testing phase, effectively assaying short-term spatial memory (Dellu et al., 1997). Sham-irradiated animals displayed normal spatial recognition by exploring the novel arm during the testing session for a significantly longer period of time than they did the start and familiar arms [Main effect of arm expl.: *F*_(__1_._73_,_15_._59__)_ = 22.56; *P* < 0.0001; *post hoc* Mult. comp: Novel vs Fam *P* < 0.001; Novel vs Start *P* < 0.01; Figure 1A]. There was a significant main effect of treatment on arm exploration within the ^1^H + ^16^O-irradiated group [*F*_(__1_._84_,_16_._57__)_ = 9.82; *P* < 0.01; Figure 1B]. Multiple comparisons analyses reveal that treated animals spent significantly more time exploring the novel than the start arm (*P* < 0.01), but failed to distinguish the novel and familiar arms, by exploring both arms for approximately equal time (*P* < 0.01), indicating a deficit in spatial memory. Discrimination ratios indicate animals’ ability to discern two objects presented in a behavioral paradigm, and can be ultimately interpreted as a measure of animals “remembering” or “forgetting” a novel object (Burke et al., 2010). Sham-irradiated animals displayed a positive discrimination ratio (μ = 0.33 ± 0.05). Irradiated animals also displayed a positive discrimination ratio (μ = 0.15 ± 0.06); however, the mean was less than half the discrimination displayed by control animals (*t* = 2.40, *P* < 0.05; Figure 1C). A measure of total distance moved was taken to ensure no bias in animal exploration due to gross locomotor deficits or other exploration-related issues. We detected no significant changes in mean total distance moved across treatment groups (*t* = 0.44, *P* = 0.67; Figure 1D).

**FIGURE 2:**
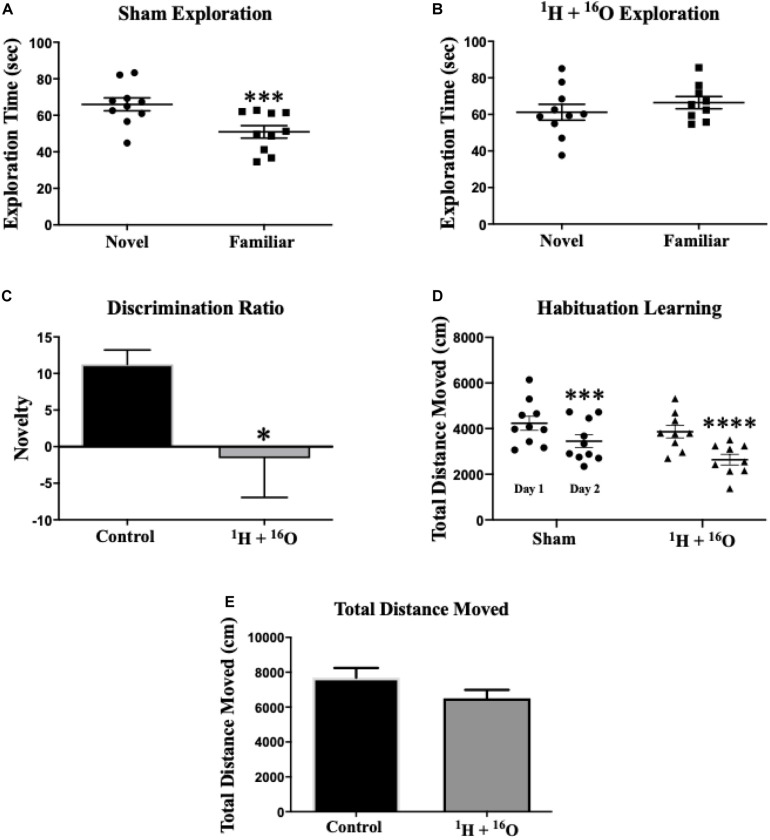
Novel object recognition. **(A)** Sham-irradiated animals spent more time exploring the novel object than the familiar one. **(B)** Radiated animals spent approximately equal time exploring both the novel and familiar objects during testing, indicating an inability to remember the familiar object. **(C)** Animals who received radiation show profoundly low object discrimination relative to sham-irradiated animals. **(D)** Sham and irradiated mice displayed normal habituation to the empty testing arena, by attenuating cumulative exploration across both habituation days. **(E)** Locomotor activity was similar across radiation groups on testing day. Average ± SEM (*n* = 10); *P < 0.05, ***P < 0.001, ****P < 0.0001.

The NOR task was used to assess non-spatial declarative object memory (Ran et al., 2016). Rodents naturally orient their head toward a novel stimulus, which provides a simple and effective method for quantifying visual recognition (Honey et al., 1998). In addition, mice rely on tactile exploration of objects in their environment, specifically under dimly-lit conditions (Hu et al., 2018). Therefore, contrasting proximity-dependent exploration of a novel versus a familiar object provides an index of object recognition and discrimination. First, the activity of the mice in the novel enclosure on training days one and two was analyzed. There was no effect of radiation on total distance moved across both habituation days [*F*_(__1_,_17__)_ = 2.50; *P* = 0.13]. Additionally, a main effect of habituation day was observed [*F*_(__1_,_17__)_ = 70.0; *P* < 0.0001], with both Sham and radiation groups resulting in significantly lower cumulative movement across habituation days (Sham: *P* < 0.001;^1^H + ^16^O: *P* < 0.0001; Figure 2D). These data indicate that mice had neither a reduced exploratory drive nor gross motor symptoms during habituation. We next assessed potential effects of radiation on habituation learning, and we observed no differences between cohorts (*t* = 1.84; *P* = 0.08; Supplementary Figure 1). Next, the training session and a test session were conducted. During the training session (day 3), mice were placed in the open-field box with two identical objects. During testing (day 4), mice were subjected to a novel and a familiar object. Movement was tracked throughout the duration of the testing session to ensure no movement-related exploration bias. No difference in mean total distance moved was detected between groups (*t* = 1.58, *P* = 0.13; Figure 2E). Statistical analysis of total exploration time during the test session revealed that exposure to ^1^H + ^16^O significantly impaired mice, as they did not show preference for the novel object (*t* = 0.82, *P* = 0.44; Figure 2B). Sham-irradiated mice showed NOR by spending more time exploring the novel than the familiar object (*t* = 5.36, *P* < 0.001; Figure 2A). Discrimination ratios showed positive object novelty discrimination in sham-irradiated animals (μ = 0.11 ± 0.02), whereas irradiated animals produced low-to-negative discrimination ratios within the SEM (μ = -0.02 ± 0.05), and the irradiated animals discrimination was significantly lower than sham-irradiated animals (*t* = 2.15, *P* < 0.05; Figure 2C).

### Dendritic Morphology

#### Dentate Gyrus Granular Cells

**FIGURE 3:**
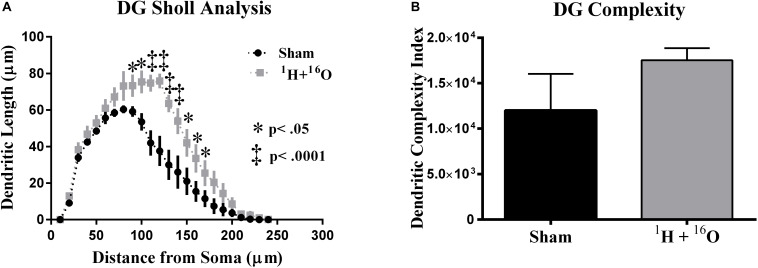
Dentate gyrus dendrite morphology. **(A)** Irradiated animals show increased dendritic length through broad distributions of the dendritic arbor. **(B)** Mean dendritic complexity was insignificantly higher in the radiation group. Average ± SEM (*n* = 5).

Sholl analyses conducted in Golgi-impregnated neurons revealed differences in dendritic morphology between treatment groups. The granular cell layer of the dentate gyrus includes neurons whose dendrites extend unilaterally out of the soma into the molecular layer (*stratum moleculare)*, receiving input from the entorhinal cortex and projecting to the Dentate polymorphic layer and *stratum lucidum* where an unmyelinated axon and its complex collateral plexus comprise mossy fibers that innervate the CA3 (Amaral et al., 2007). Our analysis, which was limited to the molecular layer dendrites, found that irradiation changed the distribution of the dendritic tree as a function of increasing 10 μm radial intervals from the soma. A significant main effect of radiation in dorsal dentate dendrites [*F*_(__1_,_8__)_ = 16.12; *P* < 0.01], as well as a significant interaction between radiation and distance from the soma [*F*_(__23_,_184__)_ = 3.40; *P* < 0.0001]. Multiple comparisons denote a significantly larger in dendritic length in irradiated animals (Fisher’s LSD: 80–90 μm, 140–160 μm, *P* < 0.05; 100–130 μm, *P* < 0.0001; Figure 3A).

To further quantify dendritic morphology, we assayed dendrite complexity (DCI), total bifurcations, dendritic ends, and dendritic length (Lom and Cohen-Cory, 1999; van der Velden et al., 2012). We observed a marked increase in total bifurcations (*t* = 2.42, *P* < 0.05; Table 1), dendritic ends (*t* = 2.82, *P* < 0.05; Table 1), and total dendritic length (*t* = 4.02, *P* < 0.01; Table 1) in irradiated animals. Despite an observed increase in nearly every parameter, we did not observe a significant increase in DCI (*t* = 1.30, *P* = 0.25; Figure 3B). However, we note that this was due to a high standard deviation in sham-irradiated animals (μ = 12,006, σ = 9,001), as compared to irradiated animals (μ = 17,528, σ = 2,970), and a low *n* of 5.

**TABLE 1:** Effects of ^1^H + ^16^O irradiation on dendrite morphology in the DG.

**Cell type and measurement**	**Sham (mean ± SEM)**	**^1^H + ^16^O (mean ± SEM)**	***P*-value**
**DG**			
Thin spines	***58.00 ± 1.15***	***62.95 ± 1.23***	***P < 0.01***
Stubby spines	28.43 ± 1.99	30.53 ± 1.13	*P = 0.35*
Mushroom spines	***15.16 ± 0.60***	***6.355 ± 0.36***	***P < 0.01***
Overall density	***24.28 ± 0.57***	***22.18 ± 0.24***	***P < 0.01***
Total dendritic length (μm)	***622.6 ± 62.97***	***919.3 ± 38.51***	***P < 0.01***
Total # branch points	***4.520 ± 0.59***	***6.160 ± 0.34***	***P < 0.05***
Dendritic ends	***6.160 ± 0.48***	***7.840 ± 0.35***	***P < 0.05***
Dendritic complexity	12006 ± 4025	17528 ± 1328	*P = 0.25*

*Radiation resulted in a marked decrease in spine density of dorsal dentate molecular layer dendrites. The shift in spine density is characterized by a minor increase in thin spine density, and major decrease in mushroom spine density in irradiated mice. Dendritic length, branch points and ends appeared to be higher in irradiated animals, despite no significant changes in dendritic complexity. Average ± SEM (*n* = 5). Bold and italic values are statistically significant.*

#### CA1 Pyramidal Neurons

**FIGURE 4:**
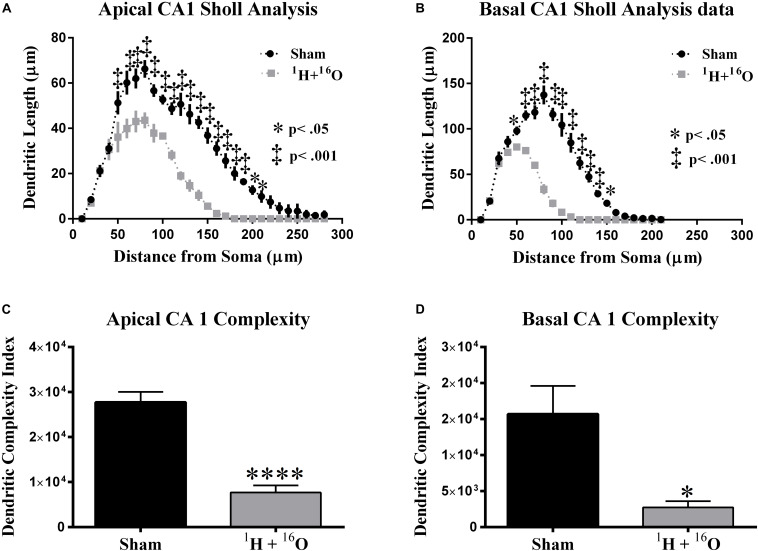
Dorsal CA1 dendritic morphology. **(A,B)** The apical and basal CA1 regions had significant dendritic length reductions through broad distances from the soma. **(C,D)** Dendritic complexity was significantly reduced as a result of treatment in both the apical and basal divisions. Average ± SEM (*n* = 5); *P < 0.05, ****P < 0.0001.

Next, we compared dendritic morphology between cohorts in the CA1 subregion. The glutamatergic hippocampal network contains pyramidal neurons in the CA subregions that branch in two distinct arbors on opposite sides of the soma. The apical side branches through the *stratum oriens*, and the basal side through the *stratum radiatum.* A two-way ANOVA detected a significant main effect of treatment between the irradiated group and sham-irradiated group in both the apical [*F*_(__1_,_8__)_ = 130.1; *P* < 0.0001] and basal [*F*_(__1_,_8__)_ = 139.9; *P* < 0.0001] divisions. Significant interactions between radiation and distance from the soma were also detected in the apical [*F*_(__27_,_216__)_ = 8.15; *P* < 0.0001] and basal [*F*_(__20_,_160__)_ = 34.91; *P* < 0.0001] CA1. *Post hoc* multiple comparisons reveal significant reductions in dendritic length far from the soma in the apical (Fisher’s LSD: 40, 90–200 μm, *P* < 0.05; 50–80 μm, *P* < 0.0001; Figure 4A) and basal (Fisher’s LSD: 40, 140 μm, *P* < 0.05; 50–130 μm, *P* < 0.0001; Figure 4B) subdivisions.

Further dendrite analysis showed a reduction in total bifurcations (*t* = 4.81, *P* < 0.01; Table 2), dendritic ends (*t* = 4.64, *P* < 0.01; Table 2), and dendritic length (*t* = 11.21, *P* < 0.0001; Table 2) in the apical division. Following suit, the basal division also showed marked reductions in bifurcations (*t* = 4.93, *P* < 0.01; Table 2), ends (*t* = 4.95, *P* < 0.01; Table 2), and length (*t* = 12.85, *P* < 0.0001; Table 2). CA1 dendritic complexity was significantly lower in both the apical (*t* = 7.21, *P* < 0.0001; Figure 4C) and basal (*t* = 3.24, *P* < 0.05; Figure 4D) regions.

**TABLE 2:** Effects of ^1^H + ^16^O irradiation on dendrite morphology in the CA1.

**Cell type and measurement**	**Sham (mean ± SEM)**	**^1^H + ^16^O (mean ± SEM)**	***P*-value**
**CA1 apical**			
Thin spines	53.85 ± 3.10	59.41 ± 1.50	*P = 0.12*
Stubby spines	***28.69 ± 1.90***	***34.37 ± 1.48***	***P < 0.05***
Mushroom spines	***17.47 ± 1.52***	***6.218 ± 0.50***	***P < 0.0001***
Overall density	22.54 ± 0.82	22.42 ± 0.35	*P = 0.89*
Total dendritic length (μm)	***774.8 ± 17.23***	***375.6 ± 31.17***	***P < 0.0001***
Total # branch points	***6.440 ± 0.31***	***4.000 ± 0.40***	***P < 0.01***
Dendritic ends	***7.440 ± 0.31***	***5.040 ± 0.41***	***P < 0.01***
Dendritic complexity	***27722 ± 2289***	***7678 ± 1575***	***P < 0.0001***
**CA1 basal**			
Thin spines	***54.99 ± 1.54***	***62.60 ± 2.08***	***P < 0.05***
Stubby spines	30.03 ± 0.66	30.91 ± 1.70	*P = 0.64*
Mushroom spines	***14.98 ± 0.95***	***6.488 ± 0.58***	***P < 0.0001***
Overall density	21.75 ± 0.73	22.23 ± 0.37	*P = 0.57*
Total dendritic length (μm)	***1110 ± 51.67***	***440.3 ± 6.835***	***P < 0.0001***
Total # branch points	***8.560 ± 1.08***	***3.080 ± 0.27***	***P < 0.01***
Dendritic ends	***11.72 ± 1.03***	***6.440 ± 0.29***	***P < 0.01***
Dendritic complexity	***15687 ± 3901***	***2748 ± 854.2***	***P < 0.05***

*The hippocampal CA1 of irradiated mice displayed altered densities of spine composition, but not in overall spine density compared to sham-irradiated animals in both apical and basal divisions. All parameters defining dendritic complexity were markedly lower in irradiated mice throughout dorsal CA1 pyramidal neurons. Average ± SEM (*n* = 5). Bold and italic values are statistically significant.*

#### CA3 Pyramidal Neurons

**FIGURE 5:**
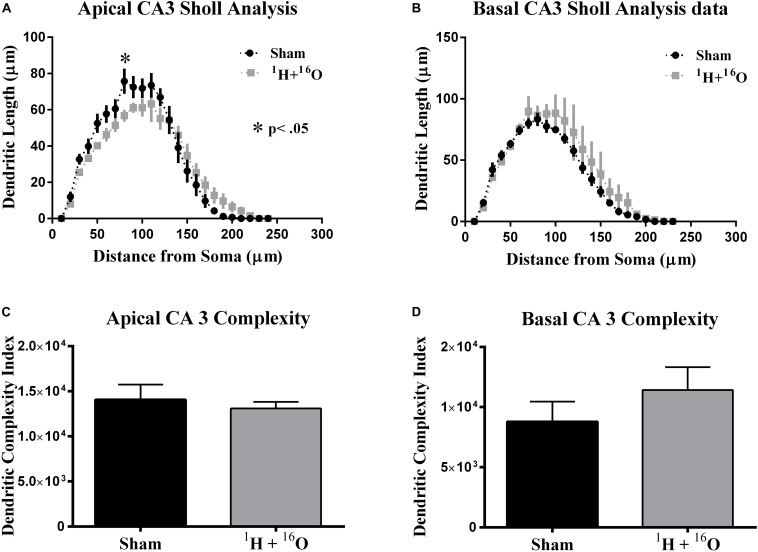
Ventral CA3 dendritic morphology. **(A)** Apical CA3 Sholl analysis reveals very minor changes to the dendritic arbor. **(B)** No significant changes in dendritic length were observed in basal CA3 pyramidal dendrites. **(C,D)** Dendritic complexity was similar in both the apical and basal subdivisions of the CA3 between cohorts. Average ± SEM (*n* = 5); *P < 0.05, ****P < 0.0001.

Our final morphological analysis was conducted in the CA3. The CA3 relays information from the DG and onto several other hippocampal subregions, the most abundant of which is the CA1, via Schaffer collateral fibers. We detected a significant interaction between radiation and distance from the soma on apical [*F*_(__23_,_184__)_ = 2.48; *P* < 0.001; Figure 5A], but not on basal dendrites [*F*_(__22_,_176__)_ = 0.65; *P* = 0.886; Figure 5B]. *Post hoc* multiple comparisons on the apical CA3 reveal only a slight decrease at 70 μm from the soma (Fisher’s LSD: *P* < 0.01) but predictably not in the basal CA3 (Figure 5B).

In accordance to our CA3 Sholl analyses, we did not observe significant changes in apical dendritic bifurcations (*t* = 0.57, *P* = 0.59; Table 3), ends (*t* = 0.86, *P* = 0.42; Table 3), and length (*t* = 0.58, *P* = 0.58; Table 3) or in basal dendritic bifurcations (*t* = 0.81, *P* = 0.44; Table 3), ends (*t* = 0.54, *P* = 0.60; Table 3), and length (*t* = 0.44, *P* = 0.67; Table 3). As expected, there were no changes in dendritic complexity due to radiation (apical, *t* = 0.52, *P* = 0.62; basal *t* = 1.04, *P* = 0.33; Figures 5C,D).

**TABLE 3:** Effects of ^1^H + ^16^O irradiation on dendrite morphology in the CA3.

**Cell type and measurement**	**Sham (mean ± SEM)**	**^1^H + ^16^O (mean ± SEM)**	***P*-value**
**CA3 apical**			
Thin spines	56.46 ± 2.76	58.34 ± 1.05	*P = 0.51*
Stubby spines	31.36 ± 2.23	34.99 ± 0.78	*P = 0.13*
Mushroom spines	***12.23 ± 0.71***	***6.673 ± 0.44***	***P < 0.0001***
Overall density	22.92 ± 0.84	21.36 ± 0.57	*P = 0.15*
Total dendritic length (μm)	753.6 ± 59.95	708.4 ± 49.30	*P = 0.5764*
Total # branch points	5.467 ± 0.49	5.160 ± 0.22	*P = 0.5855*
Dendritic ends	7.160 ± 0.58	6.600 ± 0.30	*P = 0.4157*
Dendritic complexity	14051 ± 1694	13090 ± 735.4	*P = 0.6171*
**CA3 basal**			
Thin spines	***57.23 ± 2.02***	***62.88 ± 2.01***	***P = 0.08***
Stubby spines	31.49 ± 1.90	29.85 ± 2.00	*P = 0.57*
Mushroom spines	***11.40 ± 0.47***	***7.267 ± 0.44***	***P < 0.0001***
Overall density	***22.90 ± 0.23***	***21.17 ± 0.60***	***P < 0.05***
Total dendritic length (μm)	818.8 ± 24.64	858.8 ± 86.74	*P = 0.6689*
Total # branch points	5.187 ± 0.47	5.760 ± 0.53	*P = 0.4423*
Dendritic ends	7.773 ± 0.48	8.200 ± 0.62	*P = 0.6021*
Dendritic complexity	8785 ± 1671	11422 ± 1894	*P = 0.3271*

*Mushroom spine density was lower throughout the pyramidal CA3 neurons of irradiated mice, with lowered overall spine density in the basal CA3. No significant differences were observed in overall dendrite length, branchpoint, ends and complexity between irradiated and control mice. Average ± SEM (*n* = 5). Bold and italic values are statistically significant.*

### Dendritic Spine Density and Morphology

Golgi-stained sections were also used to examine hippocampal dendritic spines. We observed a minor, but significant decrease in spine density in the dentate gyrus of the irradiated group (*t* = 3.53, *P* < 0.01; Table 1). This was accompanied by a slight increase in thin spine density (*t* = 2.90, *P* < 0.01; Table 1), and a severe reduction in mushroom spine density (*t* = 13.14, *P* < 0.0001; Table 1) in the radiation group. We did not observe differences in stubby spines (*t* = 0.96, *P* = 0.35; Table 1).

We did not observe any changes in spine density in the apical (*t* = 0.14, *P* = 0.89; Table 2) or basal CA1 (*t* = 0.59, *P* = 0.57; Table 2) subregions. The apical division revealed a slight increase in stubby spines (*t* = 2.40, *P* < 0.05; Table 2), no changes in thin spines (*t* = 1.71, *P* = 0.12; Table 2), and an explicit decrease in mushroom spines (*t* = 7.61, *P* < 0.0001; Table 2) in irradiated mice. However, differences between spines within the basal CA1 were similar to the dentate gyrus. Radiation resulted in a slight increase in thin spine density (*t* = 2.94, *P* < 0.05; Table 2), no changes in stubby spines (*t* = 0.48, *P* < 0.64; Table 2), and a substantial decrease in mushroom spines (*t* = 7.59, *P* < 0.0001; Table 2).

We then examined spine densities within the CA3. The apical region did not reveal changes in overall spine density (*t* = 1.59, *P* = 0.15; Table 3), though the basal region had a minor decrease in spine density in treated animals (*t* = 2.50, *P* < 0.05; Table 3). The apical and basal regions revealed no changes in thin (apical, *t* = 0.68, *P* = 0.51; basal, *t* = 1.97, *P* = 0.08; Table 3) or stubby (apical, *t* = 1.66, *P* = 0.13; basal, *t* = 0.59, *P* < 0.57; Table 3) spines. However, we again observed a sweeping decrease in mushroom spines of treated mice in the apical (*t* = 6.92, *P* < 0.0001; Table 3) and basal (*t* = 6.45, *P* < 0.0001; Table 3) regions of the CA3, a constant change we observed throughout the regions of the hippocampus.

### SNP Analysis of Hippocampal Lysates

**FIGURE 6:**
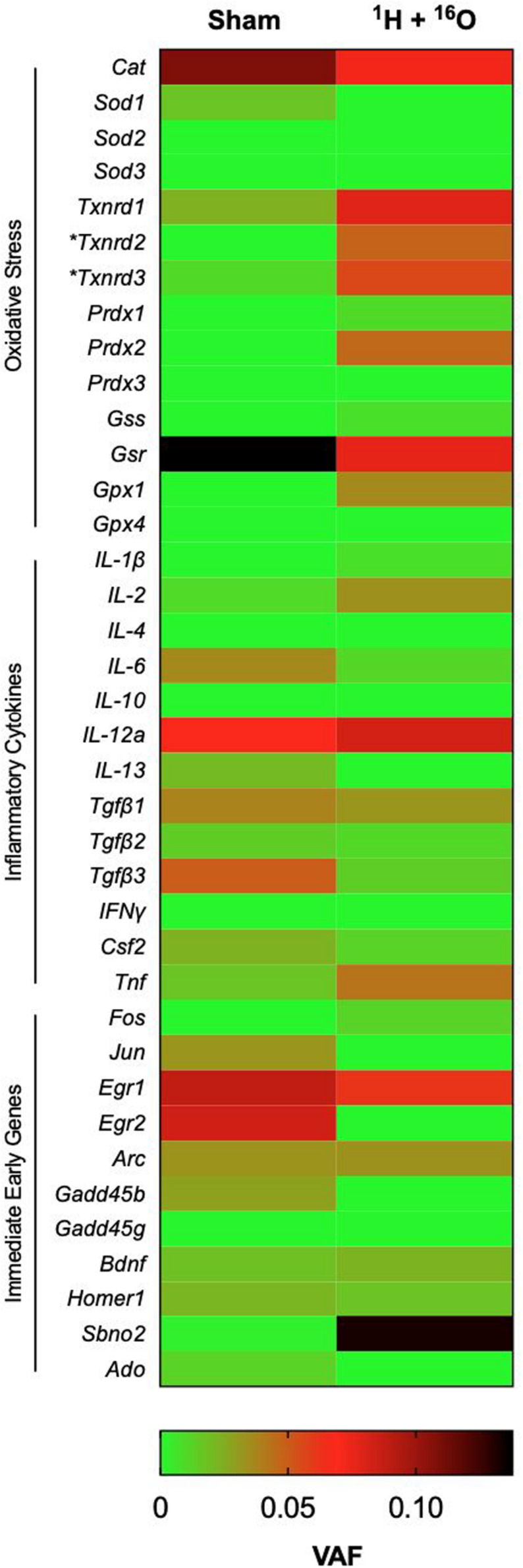
Effects of ^1^H + ^16^O on SNPs in homogenous cell populations in the hippocampus. There were no significant effects of radiation on select genes for inflammatory cytokines, oxidative stress, and immediate early genes in the whole hippocampus. The only significant radiation-dependent increase in variant allele frequency occurred on oxidative stress elements Txnrd2 and Txnrd3 loci. Mean VAF (*n* = 10); *P < 0.05.

Charged-particle radiation induces dense cores of ionization events in normal tissues, with a high mutagenic potential. We therefore expected a high number of SNPs in the DNA of homogeneous cell populations in the hippocampus even at a late time point from irradiation. We identified surprisingly few significant SNPs in immediate early genes, genes associated with oxidative stress elements, and genes involved in inflammation processes. Overall, we detected a significant difference in variant allele frequency between sham and irradiated samples in only 2 of 38 gene targets (Figure 6). The locus for thioredoxin reductase 2 was found to contain significantly higher SNPs in irradiated samples (*t* = 2.99, *P* < 0.05; Supplementary Table 2) with a mean VAF ranging from 7.9 to 9.3% in irradiated samples, and no SNPs detected in the sham group. Similarly, thioredoxin reductase 3 was also found to have a higher SNP rate, with 11.8–13.3% variant alleles (*t* = 2.53, *P* < 0.05; Supplementary Table 2). SNPs generally occurred in unique locations across various genes. In addition, we detected 6 newly identified SNPs, which occurred on every sample that met testing criteria (Supplementary Table 3 and Figure 1). Variant allele frequencies (VAF) ranged from 0 to 56%, a surprising outcome considering the heterogeneous cell population in the whole hippocampus was sequenced, but perhaps explained by the old age of the mice.

## Discussion

We investigated the effects of whole-body ^1^H + ^16^O irradiation on hippocampus-dependent behavior and dendritic morphology of mice 9 months after radiation exposure. Irradiation elicited deficits in short-term spatial and object memory, as assayed by the Y-maze and NOR tests, respectively. We observed pronounced modulation in dendritic morphology in irradiated mice as compared to sham-exposed animals. Furthermore, we observed vast reductions in mushroom spine density throughout the hippocampus.

We have previously studied the effects of ^1^H + ^16^O on the hippocampus of mice at 3 months after exposure (Kiffer et al., 2018). Mice in the present study received exposure at the same time as the aforementioned study, but were housed in a different bedding: Enrich-o’Cob, which contains small cotton nesting material mixed with the bedding. Enrich-o’Cob requires mice to find and retrieve the nesting material that is interspersed with the bedding to form a weekly nest as cages are changed, effectively cognitively enriching mice. In our previous study, we observed a deficit in short-term spatial memory as assessed by the Y-maze, similar to the present study; however, the deficit was more pronounced in the 3-month study, where irradiated mice displayed a negative discrimination ratio. The current study shows a lowered, but still positive, discrimination ratio in treated animals. Long-term housing under environmental enrichment has shown to improve cognition in rodents, and specifically, in those tested with the Y-maze (Chen et al., 2010; Hughes and Otto, 2013; Xing et al., 2016; He et al., 2017; Park et al., 2017). It is unclear whether these differences are due to time from irradiation or environmental enrichment.

Previous studies investigating the effects of whole-body exposure to ^16^O on hippocampus-dependent behavior have shown deficits in NOR. Mice exposed ^16^O showed reduced novel object exploration when tested 2–8 weeks (0.01, 0.5 Gy), and 9 months (0.05, 0.1, 0.25 Gy) postexposure (Rabin et al., 2014, 2015; Howe et al., 2019; Kiffer et al., 2019a). Six month-old transgenic Thy1-EGFP mice that received 0.3 Gy of ^16^O showed a reduced discrimination index 6 weeks after irradiation (Parihar et al., 2015). ^1^H-irradiation has also shown to induce cognitive-behavioral deficits in NOR. High-energy protons delivered to 2-month-old mice resulted in an inability to distinguish familiar and novel objects during NOR testing 3 months (0.1 Gy) after irradiation (Raber et al., 2016). Exposure to ^1^H (60%) + ^4^He (20%) + ^16^O (20%) (totaling 0.15 or 0.5 Gy) resulted in NOR deficits in male mice (Krukowski et al., 2018). We currently show a drastically reduced discrimination ratio in irradiated animals who underwent NOR testing 9-months following ^1^H (0.5 Gy) + ^16^O (0.1 Gy) exposure, despite enriched housing.

Hippocampus-dependent spatial learning and object recognition relies on the tri-synaptic circuit of the hippocampus where dentate gyrus neurons receive cortical input via the perforant pathway, relay information unidirectionally through the CA3-CA1 subregions, and transmit processed information back to the entorhinal cortex directly and via the subiculum (Moser et al., 2008; Wojtowicz, 2012; Larkin et al., 2014; Roy et al., 2017). Recent publications suggest hippocampus-dependent episodic memories require rapid synaptic plasticity in engram cells (Rubin et al., 2015; Kitamura et al., 2017). However, CA1-CA3 engrams require high place-and-context specificity and are highly dynamic, whereas DG neurons require low specificity, but are capable of maintaining a spatial code for long durations (Hainmueller and Bartos, 2018). We previously observed a pronounced increase in dendritic length and complexity of dentate gyrus mossy fibers 3 months after ^1^H + ^16^O exposure. Our current findings show that dendrites in the DG showed increased length in irradiated mice, consistent with our previous observations. The observed increase in dendritic length as a function of distribution of the arbor (90–170 μm from the soma) was nearly identical as our previous study (80–180 μm from the soma), suggesting a highly organized, non-time-dependent response to radiation. We speculate this increase in dendritic length in the DG may be a compensatory response to radiation, given the role the DG plays in encoding spatial information.

The CA1, however, suffered a drastic decrease in dendritic length and complexity, which was consistent with our previous findings, but occurred at a much wider distribution of the arbor in both the apical and basal regions. Although Golgi staining is functionally indiscriminate of specific neuronal populations due to its random impregnation, we assume the stochastic interactions between charged particles and cells suggests our findings include engram-specific neurons, though future work is needed to vet these assumptions. Interestingly, we did not observe pronounced changes in the dendrites of the pyramidal CA3 neurons, which we analyzed from the ventral region of the hippocampus. There is much debate as to whether the dorsal and ventral divisions of the hippocampus are involved in distinct processes. The dorsal hippocampus has recently been argued to be involved in cognitive information processing, whereas the ventral division is implicated in emotional and stress processing, with functionally distinct gene-expression (Fanselow and Dong, 2010; Lee et al., 2017).

Dendrites must reach respective input and output targets in order to functionally establish a computational circuit (Tavosanis, 2012). The sites where circuitry connections occur are dendritic spines, the bulbous protrusions along a dendritic shaft that host synaptosomes. The morphological characteristics of dendritic spines help dictate the stability of an individual synapse, and thus, the strength of a memory an individual synapse may be involved in maintaining (von Bohlen and Halbach, 2009). Stubby spines contain a large, bulbous head, but no defined neck. Similarly, a mushroom spine contains a large bulbous head but instead has a pronounced neck, giving its characteristic stability, and association with maintaining memories (Matsuzaki et al., 2004). Inversely, thin spines contain a thin neck, and very small bulbous, head. Thin spines are capable of maturing into mushroom spines, or disappearing altogether, and due to this plasticity, they are associated with learning (Mancuso et al., 2013). We observed pronounced reductions in mushroom spine density in all hippocampal subregions, which together with dendritic modulation, suggest radiation-induced hippocampal modulation that may be unconducive to memory processes. Notably, even subtle adjustments in morphological parameters such as branch points within pyramidal neurons, result in profound changes in firing characteristics (Ferrante et al., 2013), and individual dendrites in these neurons are arguably considered independent computational units within the broader hippocampal network (Losonczy et al., 2008).

The mutagenic potential of charged-particle radiation has been extensively studied in cell cultures and *in vivo*, though relatively little attention has been devoted to studying mutagenic effects on brain tissues (Gauny et al., 2001; Wiese et al., 2001; Yatagai, 2004; Bielefeldt-Ohmann et al., 2012; Sridharan et al., 2015). Previous *in vivo* work demonstrated that low doses of ^56^Fe particles are capable of eliciting genome-wide SNPs, which played an important role in the loss of heterozygosity in explanted kidney epithelial cells (Turker et al., 2017). Protons have likewise been observed to induce loss of heterozygosity in explanted kidney epithelial cells at higher dosages (1–5 Gy), but not at a more mission-relevant dose (0.5 Gy) (Kronenberg et al., 2009; Turker et al., 2013, 2017). Whereas the role of charged-particle radiation on epigenetic remodeling of the hippocampus has recently placed attention on the genetic influence of radiation-dependent behavioral changes, no SNP analysis has yet been conducted on hippocampal tissues (Impey et al., 2016, 2017).

High-energy charged-particles are capable of inducing complex clustered DNA lesions that preferentially result in double-strand breaks (Brenner and Ward, 1992; Asaithamby et al., 2008). The dominant repair response to such damage involves non-homologous end-joining, which has been characterized as a relatively low-fidelity repair process, and is exacerbated by the complexity of lesions (Bétermier et al., 2014). Furthermore, the phenomenon of radiation-induced delayed genomic instability raises an increased possibility of mutations at a late time point following irradiation (Limoli et al., 2000). Based on these findings, we expected hippocampal cells that survived irradiation would carry a substantial mutational load despite the relatively low dose. Our results, which focused on mutations as compared to a relatively narrow gene array found that the few identified SNPs were not radiation dependent, and are commensurate to mutagenesis associated with aging (Enge et al., 2017). We only detected a significant change in SNPs between Sham and irradiated mice on the loci for mitochondrial ROS scavengers Thioredoxin 2 and Thioredoxin 3. Interestingly, Thioredoxin 2 is a major component of the mitochondrial apoptosis-signaling pathway and glutaredoxin system, and has been found to have an increased presence in the hippocampus of aged dogs, but is lowered in the CA1 region of aged Gerbils (Tanaka, 2002; Ahn et al., 2011; Lee et al., 2015). Conversely, little has been reported of Thioredoxin 3, which appears to have low expression in the mouse brain (Sun et al., 1999). Our findings support a line of evidence suggesting that charged-particle radiation elicits aging-like pathologies, which may be dependent on oxidative stress dysregulation (Joseph et al., 1993, 1999; Casadesus et al., 2005; Poulose et al., 2011; Suman et al., 2013).

Charged-particle-induced DNA damage has been shown to occur preferentially on euchromatic regions, which is highly cell phenotype-dependent (Costes et al., 2007). In addition, the hippocampus consists of a variety of cell types, ranging from mature neurons, which are arrested at G_0_, to highly proliferating stem cell and stem cell-derived populations. To clearly establish the role of charged-particle radiation on possible polymorphisms in hippocampal tissues, future studies should include genome-wide sequencing experiments from homogeneous cell populations isolated from the irradiated hippocampus.

There is much to be learned as to how strategies currently used by astronauts, as well as other potential future countermeasures may be protective against spaceflight-related insults to the central nervous system. Physical exercise, for example appears to enhance hippocampus-dependent cognitive processes in rodents and humans (Chieffi et al., 2017). Previous rodent studies have demonstrated that antioxidant-rich diets are protective of hippocampus-dependent cognitive deficits following charged-particle exposure (Rabin et al., 2005a, b; Shukitt-Hale et al., 2013; Villasana et al., 2013; Poulose et al., 2014, 2017). There is also a question as to whether cognitive enrichment can attenuate radiation-induced cognitive deficits. One study assessed whether rats that underwent the familiarization phase of NOR 2–4 h prior to exposure to either 25cGy of ^56^Fe or 5 cGy of ^16^O, and then were tested 24 h later, would have better recognition of the novel object, than rats that underwent familiarization 24 h and testing 48 h after radiation. In both cases, acute object recognition deficits occurred only in the group that underwent familiarization prior to irradiation (Rabin et al., 2015). However, a recent study involving an appetitive behavioral platform that depends on 1 month of near daily touchscreen training found that mice receiving 3 6.7 Gy fractions of ^56^Fe improved hippocampus-dependent pattern separation on a number of tasks. This was also true of non-trained mice undergoing contextual fear conditioning 2 months following exposure to either ^56^Fe or ^28^Si, though it is not known if improvements in performance of such tasks is pathological behavior (Whoolery et al., 2020). Although our experiment, which to our knowledge is the first to test whether housing enrichment prior to, and after radiation would prevent hippocampal sequelae, suggests enrichment on its own was not sufficient to fully rescue radiation-induced deficits. It is important to assess whether multiple factors such as diet, exercise and enrichment can together, prevent radiation-dependent alterations.

Mounting evidence highlights cognitive hazards associated with spaceflight. We show that a Mars-mission-relevant exposure to ^1^H followed by ^16^O an hour later results in object and short-term spatial memory deficits 9 months after exposure. Hippocampus-wide neuronal morphology alterations were observed in combination with drastic reductions in mushroom spine density. Surprisingly, radiation did not elicit noticeable widespread SNPs across select gene targets in the hippocampus when compared to sham-irradiated samples, with the exception of a higher VAF on loci for thioredoxin reductase 2 and 3 in irradiated samples. Future studies should elucidate mechanisms of dendrite dysmorphia in individual hippocampal subregions and address whether changes in dose-rate induce the same effects as single exposures. Future studies should also elucidate mutation rates on distinct cell populations in the hippocampus.

## Data Availability Statement

The datasets presented in this study can be found in online repositories. The names of the repository/repositories and accession number(s) can be found below: https://www.ebi.ac.uk/ena/data/view/PRJEB38379.

## Ethics Statement

The animal study was reviewed and approved by University of Arkansas for Medical Sciences Animal Welfare Assurance number A3063-01.

## Author Contributions

AA and MBo: conceptualization, resources, and funding acquisition. FK: behavioral testing, DNA sequencing, and writing—original draft preparation. FK and TM: DNA isolation. TA, JA, and TG: software monitoring for behavioral testing. FK, TA, JA, and TG: Golgi staining. FK and AA: statistical analyses. JW: histological analyses. VS: animal husbandry and management. FK, TA, JA, TG, and VS: animal sacrifice and tissue collection. MBa and FK: DNA sequencing data curation. All authors contributed to the article and approved the submitted version.

## Conflict of Interest

The authors declare that the research was conducted in the absence of any commercial or financial relationships that could be construed as a potential conflict of interest.
