# Supplementary material for: Late Effects of 1H + 16O on Short-Term and Object Memory, Hippocampal Dendritic Morphology and Mutagenesis
Source: Front Behav Neurosci. 2020 Jun 26;14:96. doi: 10.3389/fnbeh.2020.00096 (PMC7332779; doi:10.3389/fnbeh.2020.00096)

*Supplementary Materials*

**Table 1.** Custom gene target panel, gene locations, and amplicons generated. Gene targets were immediate early genes, oxidative stress elements, and genes associated with inflammation.

**
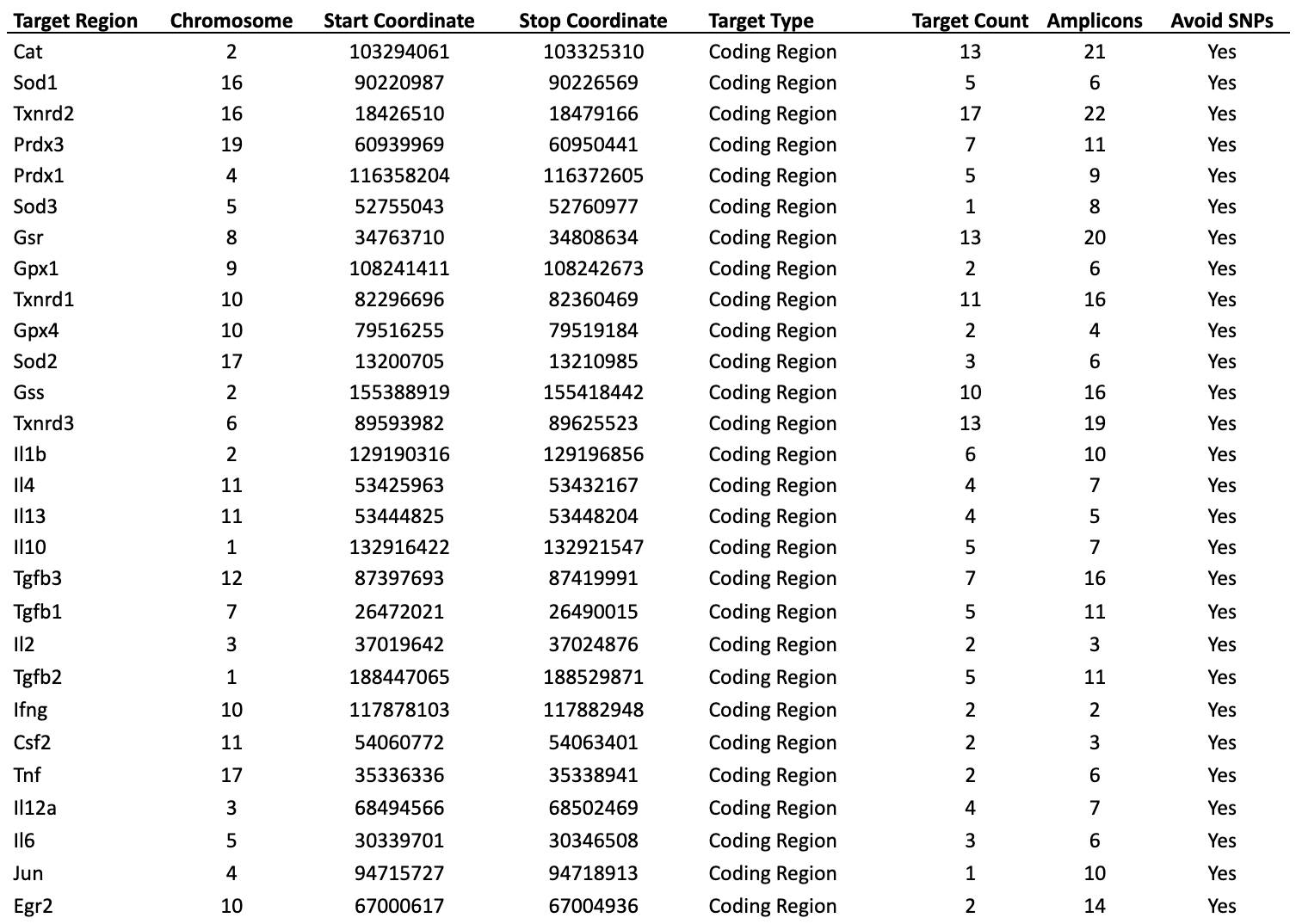
**

**
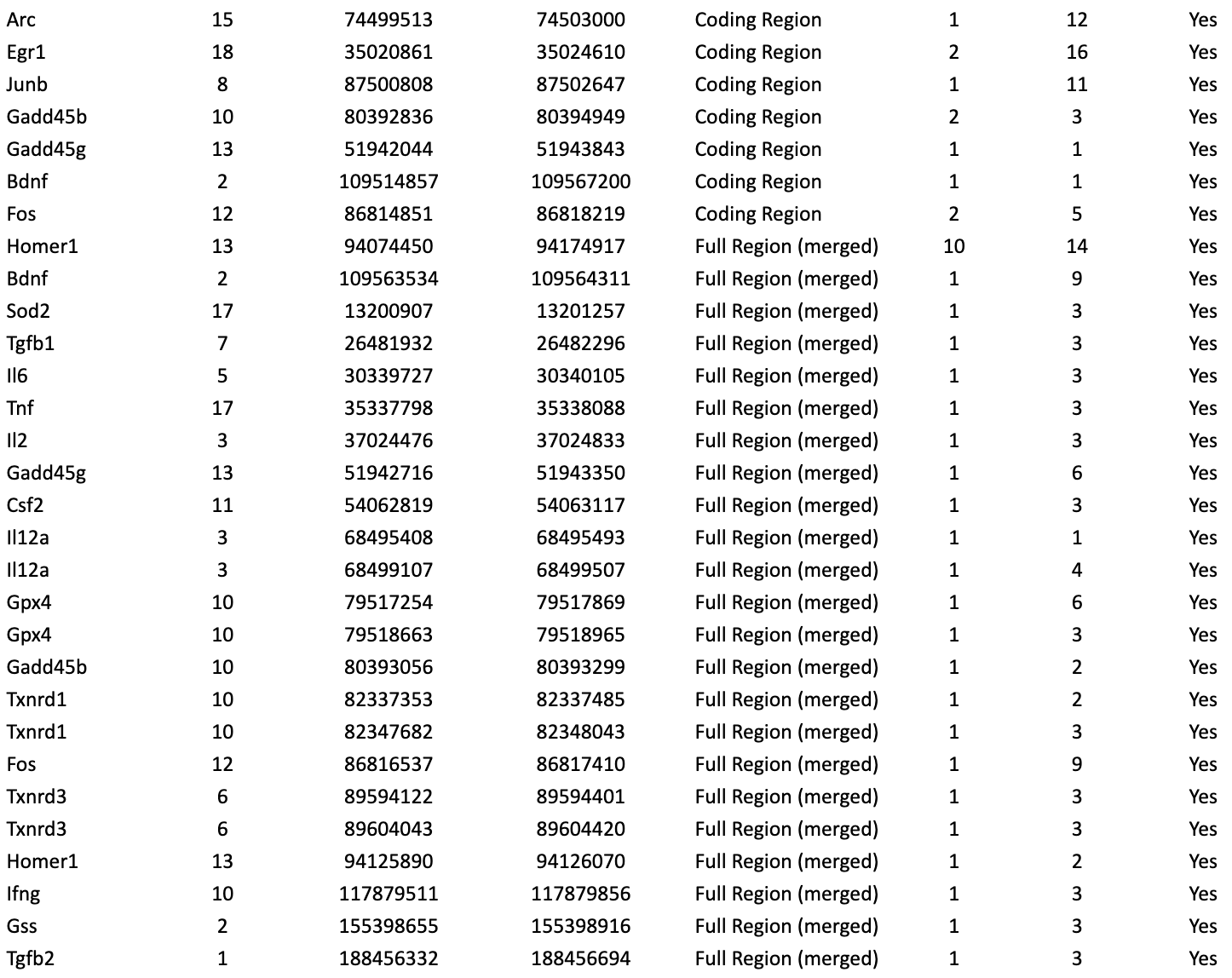
**

**Table 2.** Variant allele analyses for all gene targets


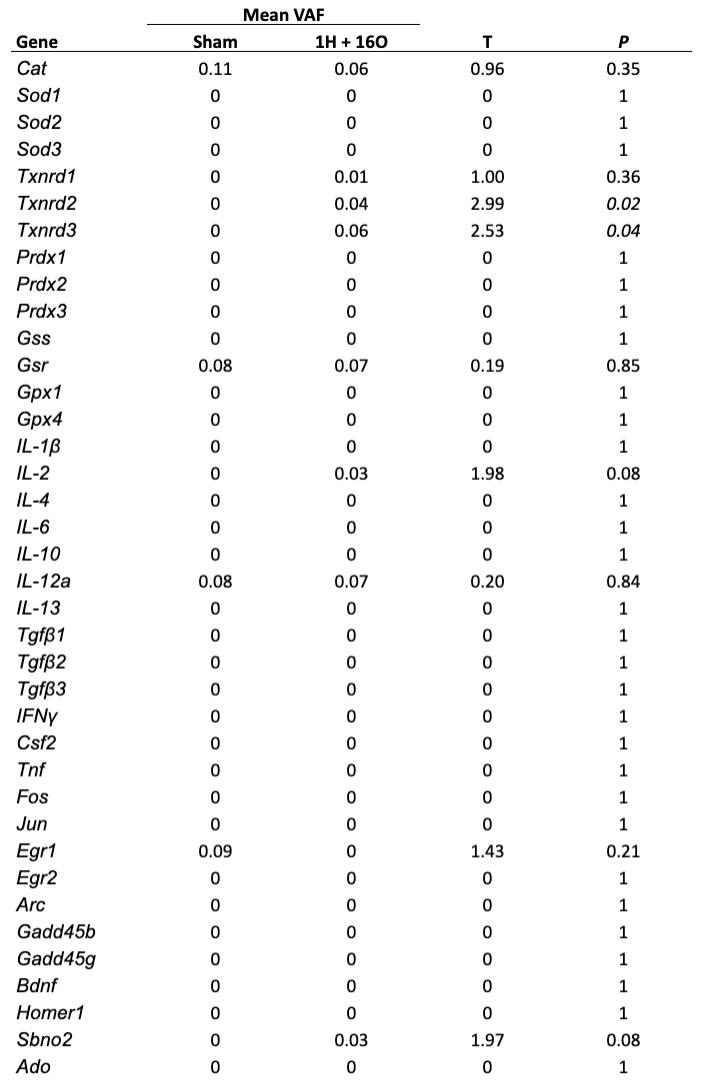


**Table 3**. Novel identified SNPs occurring on all, or the majority of tested samples.
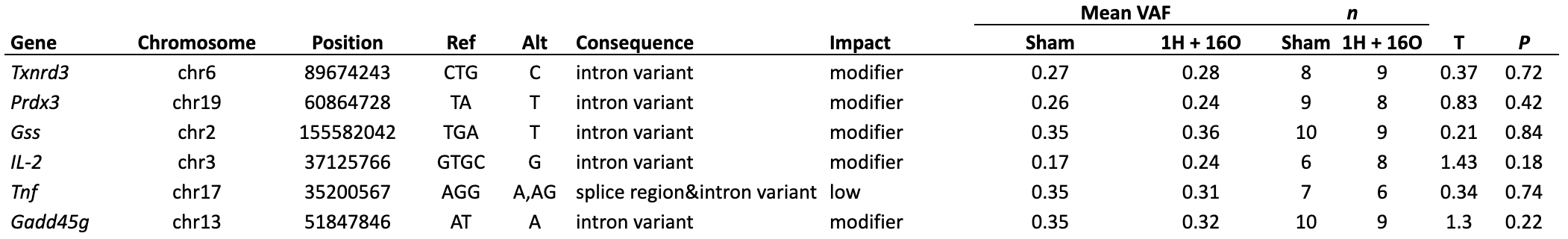


**Fig 1.** Heat map for novel identified SNPs occurring on all or the majority of samples.

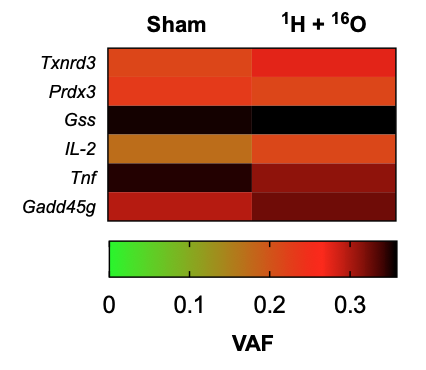

Supplement: Supplementary file 1 [file Table_1.DOCX]
